# Recurrent Left-Sided Hepatic Hydrothorax Leading to Liver-Mediated Dyspnea

**DOI:** 10.7759/cureus.63180

**Published:** 2024-06-26

**Authors:** Dhaval Trivedi, Kin Li, Sana Ahmed, Franklyn Fenton, Saleem Shahzad

**Affiliations:** 1 Internal Medicine, NewYork-Presbyterian Brooklyn Methodist Hospital, New York City, USA; 2 Pulmonary and Critical Care Medicine, NewYork-Presbyterian Brooklyn Methodist Hospital, New York City, USA

**Keywords:** pleural effusion, left sided, cirrhosis, ascites, hepatic hydrothorax

## Abstract

Cirrhosis is a common liver condition caused by several etiologies including alcohol use disorder, infectious hepatitis, and metabolic dysfunction associated with liver disease. Although common symptomatic complications of cirrhosis include malaise, gastrointestinal bleeding, and abdominal distension, shortness of breath is a less common phenomenon that may occur. Hepatic hydrothorax (HH) is an uncommon cause of shortness of breath that is believed to be caused by the accumulation of ascitic fluid in the pleural space. While most cases of HH occur with ascites and the right side, we hereby present a case of a 70-year-old female with left-sided HH without ascites.

## Introduction

Dyspnea is a common pulmonary complication that can be caused by pulmonary or cardiac diseases such as heart failure, pneumonia, or chronic obstructive pulmonary disease. While many other conditions such as anemia or mental disorders may present similarly, hepatic causes of shortness of breath are uncommon [1]. Hepatic hydrothorax (HH) describes the presence of greater than 500 milliliters of fluid in the pleural space as sequelae of liver dysfunction in the absence of renal, cardiac, and pulmonary pathologies. This type of presentation is usually noted in the setting of decompensated liver failure alongside portal hypertension and ascitic fluid accumulation. Although most cases of HH typically present with ascites, some cases may present more insidiously without typical signs of ascites [2].

Previous studies have reported incidence rates of HH in the setting of cirrhosis and portal hypertension range between 5% and 16%. In previous retrospective case series, patients with HH typically presented with a right-sided pleural effusion (73%). However, left-sided (17%) and bilateral pleural effusions (10%) also occurred infrequently. In the majority of these cases, ascites were also a commonly associated finding with only some case series reporting 9% of patients without any ascites [3,4]. However, left-sided HH without ascites is rarely reported. In this case presentation, we report a 70-year-old female who presented with left-sided HH without any ascites.

## Case presentation

A 70-year-old female with a history of hypertension, type 2 diabetes mellitus, treated hepatitis C (genotype 3a), metabolic dysfunction-associated steatotic liver disease (MASLD), and cirrhosis associated with grade 1 esophageal varices presented to the Emergency Department with complaints of abdominal pain and leg swelling for one week with new-onset shortness of breath for three days. The abdominal pain was epigastric, prandial, and positional with radiation to her right upper quadrant. The pain was unrelieved with acetaminophen. The shortness of breath was previously only associated with exertion, but now occurs at rest.

**Figure 1:**
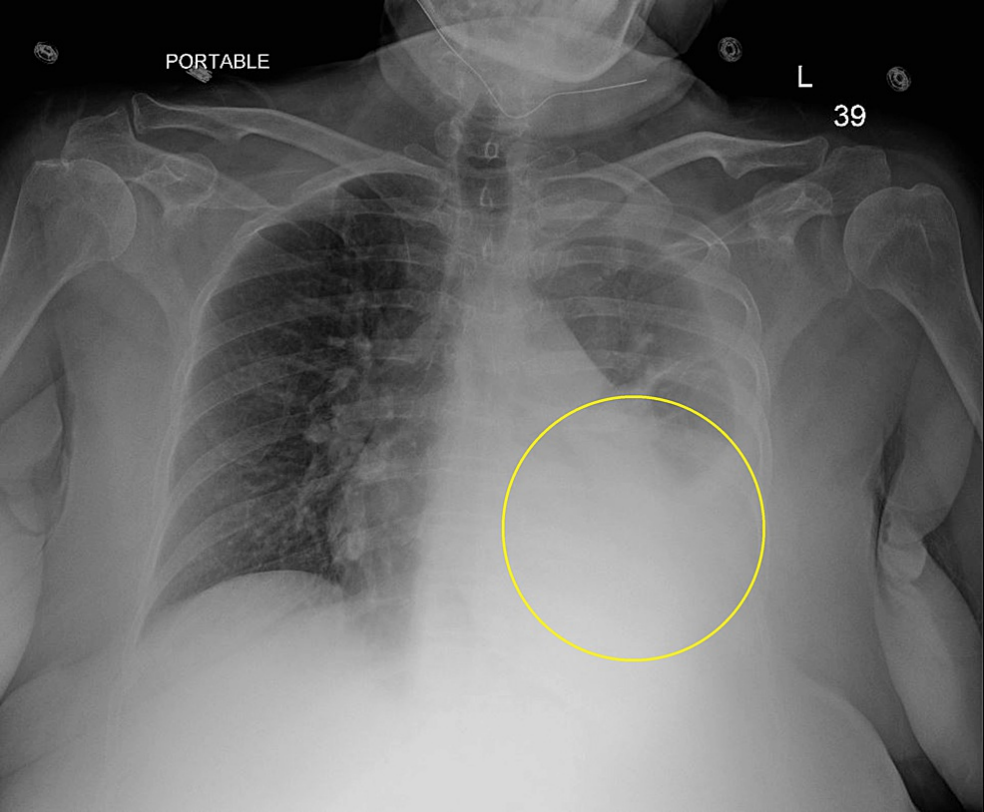
Chest X-ray Yellow circle: pleural effusion

**Figure 2:**
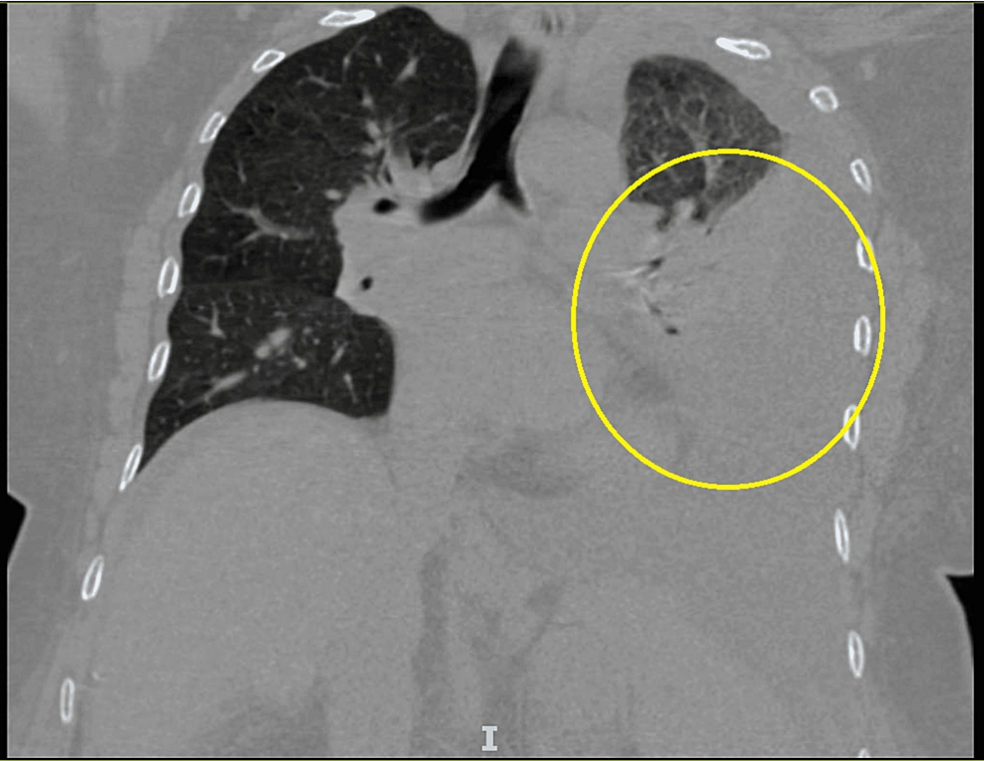
CT Chest Yellow circle: pleural effusion

In the Emergency Department, her vital signs were normal, and physical examination revealed epigastric tenderness, lower extremity non-pitting edema, and decreased breath sounds in the left lower lung field. An initial chest X-ray (CXR) was performed which revealed a left-sided pleural effusion (Figure 1, yellow circle). Initial labs (Table 1) were remarkable for leukopenia, thrombocytopenia, hypokalemia, hypoalbuminemia, hyperbilirubinemia, transaminitis, and elevated prothrombin time (PT)/international normalized ratio (INR). CT abdomen and pelvis revealed a nodular contour of the liver, ​​splenomegaly with multiple prominent vascular collaterals, moderate ascites, and gastroesophageal varices (Figure 2, yellow circle).

**Table 1:** Initial Laboratory Values (Complete Blood Count, Complete Metabolic Panel, and Coagulation Profile)

Laboratory Value	Patient's Value	Reference Range
White Blood Cell	3.2	3.5-11.0 x 10^3/uL
Hemoglobin	13.4	11.5-14.5 g/dL
Platelets	58	150-400 x 10^3/uL
Sodium	137	136-145 mmol/L
Potassium	3.3	3.5-5.1 mmol/L
Chloride	103	98-107 mmol/L
Carbon Dioxide	23	22-29 mmol/L
Blood Urea Nitrogen	10	8-23 mg/dL
Creatinine	17	0.51-0.95 mg/dL
Glucose	0.57	74-106 mg/dL
Aspartate Transaminase	79	<40 U/L
Alanine Transaminase	28	<40 U/L
Alkaline Phosphatase	112	<200 U/L
Total Bilirubin	3.4	0.1-1.2 mg/dL
Direct Bilirubin	0.6	0.0-0.3 mg/dL
Plasma Albumin	2.7	3.5-5.2 g/dL
Activated Partial Thromboplastin Time	37.9	25.1-36.5 seconds
Prothrombin Time	19.3	9.4-21.5 seconds
International Normalized Ratio	1.7	0.9-1.2 ratio

As seen in Table 1, initial labs were remarkable for leukopenia, thrombocytopenia, hypokalemia, hypoalbuminemia, hyperbilirubinemia, transaminitis, and elevated PT/INR. CT abdomen and pelvis revealed nodular contour of the liver, ​​splenomegaly with multiple prominent vascular collaterals, moderate ascites, and gastroesophageal varices (Figure 2). Abdominal ultrasound was performed for the evaluation of ascitic fluid evaluation, but low volume anechoic free fluid was found.

**Figure 3:**
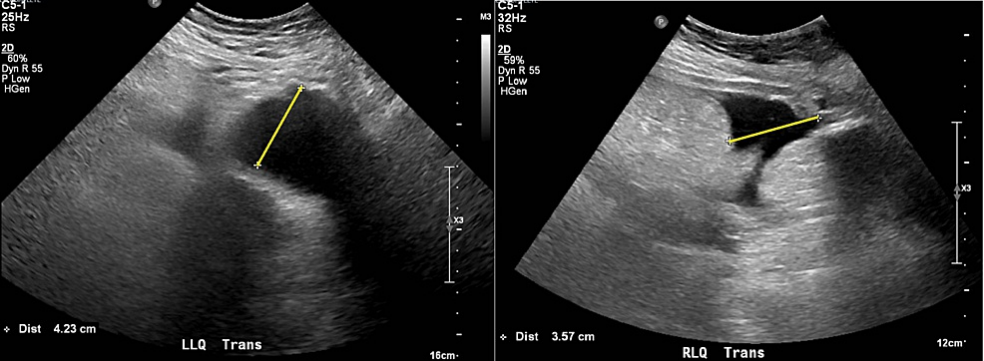
Abdominal Ultrasound for Ascites Evaluation LLQ trans: left lower quadrant transabdominal; RLQ trans: right lower quadrant transabdominal

However, abdominal ultrasound was performed for the evaluation of ascitic fluid but only revealed low-volume free fluid (Figure 3, yellow lines) with a demonstration of large left pleural effusion. Given the concern for new-onset heart failure, a transthoracic echocardiogram was ordered, which revealed a normal systolic ejection fraction. Additionally, as the patient met sepsis criteria, a septic workup was performed and ceftriaxone and azithromycin were initiated for community-acquired pneumonia coverage. Septic workup was found to be negative with normal procalcitonin and antibiotics were subsequently stopped.

Due to persistent shortness of breath, diagnostic and therapeutic thoracentesis was performed. Post-procedure CXR revealed persistent but decreased left-sided pleural effusion. The culture and cytology on the fluid sample were unremarkable. The fluid protein was noted to be 2.2 g/dL and the fluid lactate dehydrogenase was 86 g/dL. Analysis using Light's criteria revealed the sample to be transudative, as both the fluid-to-serum protein ratio and the fluid-to-lactate dehydrogenase ratio were less than 0.5 and two-thirds of the upper limit of normal, respectively (Table 2).

**Table 2:** Initial Diagnostic Thoracentesis

Laboratory Value	Patient's Value	Transudative Reference Range
Fluid/Serum Protein (g/dL)	2.0/6.6	< 0.5
Fluid/Serum Lactate Dehydrogenase (U/L)	86/287	< ⅔ the upper limit of normal serum

The following day, CXR was once again repeated due to shortness of breath complaint, which revealed increased left-sided pleural effusion. Repeat diagnostic and therapeutic thoracentesis was performed with subsequent SPAG (serum pleural-ascites gradient) studies (Table 3). The serum albumin was 2.2 g/dL and the fluid albumin was 1.058 g/dL, which revealed a SPAG of 1.142 g/dL. SPAG studies revealed cirrhosis as an etiology for effusion. Technetium-99m sulfur colloid nuclear scan was unavailable for nuclear evaluation for HH. Given the concern for HH as an etiology for pleural effusion, diuresis therapy was initiated with spironolactone 100 mg daily and furosemide 40 mg twice daily, which provided symptomatic relief. Ultimately, the patient was discharged with spironolactone 200 mg daily and furosemide 40 mg twice daily, which was subsequently halved at an outpatient hepatology visit. One month post-discharge and initiation of diuresis, repeat CXR showed complete resolution of pleural effusion.

**Table 3:** Repeat Diagnostic Thoracentesis

Laboratory Value	Patient's Value	Transudative Reference Range
Serum Albumin	2.2	3.5-5.2 g/dL
Fluid Albumin	1.058	<1 g/dL

## Discussion

In patients presenting with HH, mortality is high with some case series indicating a 57% mortality rate within 12 months and 4-5 months in those with refractory cases [2]. In cases where patients undergo transjugular intrahepatic portosystemic shunt (TIPS), mortality extends to 845 days, and those with transplants indicate the longest survival rates [3].

HH is typically a diagnosis of exclusion in which underlying cardiopulmonary or renal disease has been excluded. Although most patients with HH may have cirrhosis and ascites, some patients may present with HH as the first sign of liver failure and without ascites. In our patient, she had known cirrhosis without a history of ascites. While her initial CT of the abdomen and pelvis showed large pleural effusion and moderate ascites, there was only minimal free fluid in the abdomen on further abdominal ultrasonography.

The exact mechanisms for the development of HH are not well known. However, several theories have been postulated including hypoalbuminemia, structural defects, and osmotic and hydrostatic pressure differentials within the azygos and lymphatic systems [5]. The most accepted theory is the direct passage of ascitic fluid from the peritoneal to the pleural cavity through diaphragmatic defects, which are referred to as pleuroperitoneal communications. Predominantly occurring on the right side, due to the embryological development of the diaphragm, these microscopic defects have been described as discontinuities of collagen within the tendinous portion of the diaphragm [6]. These defects have been classified into four morphological types of various severity including type 1 with no obvious defect, type 2 with blebs within the diaphragm, type 3 with broken defects or fenestrations in the diaphragm, and type 4 with multiple gaps in the diaphragm [7].

Diagnosis of HH typically involves a combination of imaging studies and pleural fluid testing. HH is usually first seen on chest radiographs. In patients with suspected HH, a thoracentesis is typically performed for fluid analysis. HH typically will have a transudative effusion based on Light’s criteria. However, the use of serum-pleural albumin gradient has also been suggested as another diagnostic criterion for HH as some studies showed all patients with HH had SPAG measuring >1.1 g/dL [8]. An echocardiogram should also be performed to rule out any cardiac etiologies and abdominal ultrasounds to assess the liver, portal and hepatic veins, ascites, and kidneys. In cases where the diagnosis of HH is uncertain, intraperitoneal injection of radioisotopes of 99mTc-human serum albumin may demonstrate migration from the peritoneal cavity into pleural space and confirm the diagnosis of HH. The most optimal timing for this study is typically performed after therapeutic thoracentesis.

The management of HH is nuanced. In those with an initial diagnosis of HH, treatment should be focused on eliminating the cause of ascites. The use of sodium restriction and the use of diuretics are typically a mainstay in the initial management of ascite reduction [9]. In refractory HH, or roughly 21-26% of medically treated patients, the only definitive treatment is liver transplantation [9,10]. While awaiting transplantation, the goal is to reduce the symptomatic burden and the prevention of pulmonary complications through interventions such as diuresis, thoracentesis, chest tube placement, pleural catheters, TIPS, and surgical interventions (pleurodesis, diaphragmatic repair, or pleural-venous shunting) [11,12]. Our case demonstrated a case of recurrent atypical left-sided hydrothorax without ascites, which was initially treated with aggressive diuresis and sodium restriction. Although our patient had a resolution of HH with mainstay therapy, consideration for liver transplantation was and should be further discussed in the multidisciplinary approach to improve long-term outcomes in patients' mortality and morbidity.

## Conclusions

In conclusion, HH remains a challenging complication of liver disease with a high mortality rate, particularly in refractory cases. The condition often requires a nuanced approach to diagnosis and management, incorporating imaging studies, fluid analysis, and exclusion of other potential causes. Initial management typically involves sodium restriction and diuretics, while definitive treatment for refractory cases hinges on liver transplantation. The case discussed underscores the complexity of HH, highlighting the need for a multidisciplinary approach to optimize patient outcomes, reduce symptomatic burden, and improve survival rates through advanced interventions such as TIPS and liver transplantation.
